# Serotonin transporter gene polymorphisms & obsessive-compulsive disorder

**Published:** 2010-12

**Authors:** Humberto Nicolini

**Affiliations:** Servicios de Atención Psiquiátrica Minister of Health, México

Obsessive compulsive disorder (OCD) is a neuropsychiatric condition that has been shown to be heritable and for which a major gene effect has been reported based on segregation studies^1^,^2^. However, linkage analysis studies have not been able to confirm a locus for OCD^3^. The most promising candidate genes for OCD at the present time are in the serotonin system and the glutamate system^4^. The *SCL6A4* gene has been the most extensively studied functional polymorphism in psychiatry, providing worldwide evidence of its importance in several disorders, such as OCD.

The efficacy of selective serotonin reuptake inhibitors (SSRIs) in the treatment of OCD has lead to the hypothesis of a serotonergic dysfunction in this disorder^5^. Since SSRIs acts on the serotonin (5HT) transporter (5HTT), it has been suggested that the 5HTT gene (*SCL6A4*), located on chromosome 17q12^6^, could be a good candidate for OCD^7^–^9^. A functional polymorphism in the promoter region has been described^10^. This 5HTT-linked polymorphic region (5HTTLPR) is situated in a GC-rich region composed of 20-23 bp repeat units. The biallelic polymorphism consists of an insertion (long allele, “1”) or a deletion (short allele, “s”) of 44 bp. The serotonin transporter (SERT) is probably the most widely studied gene in psychiatry^11^. Previous studies have reported the “l” allele is as characterized by increased transcriptional activity as well as increased basal re-uptake of 5-HT *in vitro* compared with the short form of the 5HTTLPR^10^.

A possible association between 5HTTLPR polymorphism and OCD has been studied by some research groups. Billett *et al*^7^ could not find an association in 72 OCD patients compared to 72 controls. However, a relative increase (not statistically significant) of homozygous (“l” allele) in the OCD group was observed. Bengel *et al*^8^, reported that OCD patients were more likely to carry two copies of the long allele (“l”), compared to controls. A study by McDougle *et al*^9^ using a family-controlled transmission disequilibrium test (TDT), reported a predominant “l” allele transmission in 24 heterozygous parents to their OCD affected sibs. A preferred “l” allele transmission was also observed in 10 out of 13 SSRI non-responders. In addition, an analysis of *SCL6A4* coding region has not revealed differences in OCD patients^12^. As suggested by these studies of a possible relationship between OCD and the “l” allele, our group analyzed the 5-HTTLPR polymorphism in Mexican OCD patients using a case-control methodology. Besides, we analyzed alternative methods that employ family-based sampling to minimize the effects of population heterogeneity in a sample of 43 trios^13^. Our results did not show a positive association between this variant and Mexican OCD patients. However, previous studies showed an association between OCD and “l” allele of 5-HTTLPR polymorphism, although the literature still remains controversial^8^,^13^–^15^. In the case of Hu *et al*^14^, a single nucleotide polymorphism (SNP) that converts the long allele to a functionally short one was important in determining the significance of the SERT gene in OCD. The long allele containing the A variant of the SNP was associated with OCD, while the long-G and short alleles were not. Thus other groups should investigate this SNP in their analyses.

In addition, many association studies have resulted in controversial results because population stratification cannot easily be avoided^16^. Later studies showed that allele frequencies of the 5HTTLPR could vary widely between populations. The allele frequencies of our control group^13^ were similar to Caucasian and European-American populations previously reported^8^,^17^. These frequencies differ from those reported for Japanese and African-American subjects^18^. Since use of family-based approaches like TDT and HHRR may overcome ethnic stratification, analyzing a sample of trios for confirmation of an association between OCD and the *SCL6A4* gene should always be performed. Family-based association analysis confirmed negative linkage disequilibrium between the 5HTTLPR polymorphism and OCD. TDT and HRR analysis did not reveal preferential “l” allele transmission. These studies did not support McDougle’s initial hypothesis^9^that the “s” allele would be associated with increased susceptibility to OCD. This hypothesis was based on the report of an association between the “s” allele and neuroticism^17^and confirmed later in the susceptibility to depression in interaction with life events^19^. Neuroticism shares common genetic origins with affective and anxiety disorders, with each of many genes thought to contribute small amounts of variance^20^. Also, an association between 5HTTLPR and some disorders such as depression, life events and depression in psychosis and seasonal affective disorder and seasonality has been observed^21^–^24^. All these findings, relating the “s” allele with some psychiatric disorders, may agree with the 5HT-anxiogenic theory and with the SSRI mechanism of action in these disorders as proposed by Lesch *et al*^17^. Moreover, if the expression of 5HTT is reduced in carriers of the “s” allele, the same dose of SSRI could increase synaptic 5-HT and induce desensitization of several postsynaptic 5-HT receptors. Since the evidence may support the hypothesis of a relationship between the “s” allele and anxiety-related personality traits, studies of phenotypes based on personality traits in OCD combined with the analysis of 5HTTLPR polymorphism may provide an important tool in disentangling genetic factors involved in the disorder. Extensive family-based studies combined with population genetic studies are needed, from large, and carefully collected samples using various phenotype definitions such as co-morbidity, age of onset, type of obsessions and compulsions, and personality an temperament dimensions among others before reaching a more definitive conclusion.

New evidence showing that some of the previous results may differ since the involvement of the 5HTTLPR (A/G) gene polymorphism modulating the 5HTTLPR, it may impose to new studies the need to assess this polymorphism. In addition, most association data come from case-control studies that do not assess population stratification instead of family based samples. The study presented in this issue by Tibrewal *et al*^25^, addresses both methodological concerns. It evaluates population stratification as well as the modulating 5HTTLPR (A/G) gene polymorphism. Due to small sample size it is difficult to see an effect of common genes of minor effects. Probably, some of the rare gene variants (CNVs) in these common alleles may have a larger effect in disease aetiology. However, it is intriguing to find a modest statistically significant association of the dominant model of 5HTTLPR (A/G) (non-risk allele: LA; risk alleles: SA, SG, LG) with the severity index or YBOCS score. These data may support the idea that the 5-HT system is altered in the brain of OCD patients depending upon severity. This sub-sample (including the ethnic component) may provide a great opportunity to further explore the contribution of rare variants by methods of deep sequencing. If the peripheral findings translate into the brain, increased 5-HT uptake or storage in the presynaptic terminal would be expected to diminish extracellular 5-HT availability. Unfortunately, a direct assessment of synaptic 5-HT in patients with OCD is not possible with present technologies. However, clinical pharmacological data may also provide additional phenotypes to better understand the participation of 5-HT in OCD genetic risk.
